# Development and Characterization of Functional Starch-Based Films Incorporating Free or Microencapsulated Spent Black Tea Extract

**DOI:** 10.3390/molecules26133898

**Published:** 2021-06-25

**Authors:** Surakshi Wimangika Rajapaksha, Naoto Shimizu

**Affiliations:** 1 Laboratory of Agricultural Bio-System Engineering, Graduate School of Agriculture, Hokkaido University, Hokkaido 060-8589, Japan; surakshi.wima@gmail.com; 2 Research Faculty of Agriculture/Field Science Center for Northern Biosphere, Hokkaido University, Hokkaido 060-8589, Japan

**Keywords:** biodegradable, edible film, release, polyphenols, antioxidant activity, pectin, sodium caseinate, oil oxidation, waste biomass

## Abstract

Antioxidant polyphenols in black tea residue are an underused source of bioactive compounds. Microencapsulation can turn them into a valuable functional ingredient for different food applications. This study investigated the potential of using spent black tea extract (SBT) as an active ingredient in food packaging. Free or microencapsulated forms of SBT, using a pectin–sodium caseinate mixture as a wall material, were incorporated in a cassava starch matrix and films developed by casting. The effect of incorporating SBT at different polyphenol contents (0.17% and 0.34%) on the structural, physical, and antioxidant properties of the films, the migration of active compounds into different food simulants and their performance at preventing lipid oxidation were evaluated. The results showed that adding free SBT modified the film structure by forming hydrogen bonds with starch, creating a less elastic film with antioxidant activity (173 and 587 µg(GAE)/g film). Incorporating microencapsulated SBT improved the mechanical properties of active films and preserved their antioxidant activity (276 and 627 µg(GAE)/g film). Encapsulates significantly enhanced the release of antioxidant polyphenols into both aqueous and fatty food simulants. Both types of active film exhibited better barrier properties against UV light and water vapour than the control starch film and delayed lipid oxidation up to 35 d. This study revealed that starch film incorporating microencapsulated SBT can be used as a functional food packaging to protect fatty foods from oxidation.

## 1. Introduction

Lipid oxidation or oxidative rancidity, one of the main causes of food deterioration alongside microbial spoilage, generates harmful compounds in foods as well as degrading their colours and nutrients [1]. Synthetic antioxidants such as butylated hydroxyanisole (BHA), butylated hydroxy toluene (BHT), and propyl gallate have often been used to overcome rancidity. However, undesirable side-effects associated with these synthetic antioxidants have directed scientific studies towards investigating natural antioxidants [2,3]. Therefore, phenolic compounds originating from plants, the main category of natural antioxidants, have become of growing scientific interest.

The large amount of waste arising from food manufacturing is a potential source of underused polyphenols which possess antioxidant power [4,5]. In this sense, using this waste is a sustainable and economically attractive way of obtaining natural antioxidants.

Tea has been a popular beverage since ancient times. One type, black tea, is consumed throughout the world and contains many types of polyphenols such as theaflavin, thearubigin, and catechin which are responsible for its antioxidant activity [6,7]. Tea is prepared by infusing tea leaves in boiling water for a short time. After black tea is brewed during the industrial beverage manufacturing process, the remaining residue, spent black tea (SBT), is usually discarded as waste. SBT still contains a significantly high and recoverable quantity of antioxidant phenolic compounds and our previous study has shown that subcritical water extraction with ethanol as a co-solvent is an efficient process for extracting phenolic compounds from SBT [8]. This method, subcritical solvent extraction (SSE), uses a pressurized mixture of water and ethanol in the liquid state above its boiling point to enhance the solubility of the compounds and the mass transfer rate during the extraction process, thereby improving the extract yield while decreasing the time and solvent consumption during the process.

The phenolic extract from SBT can be incorporated directly into foods or in food packaging to control lipid oxidation. However, adding phenolic extracts directly to food may neutralize their antioxidant activity after they react with the food and reduce its quality. As the lipid oxidation process is induced from the surface of the foodstuff, designing a functional food film incorporating phenolic extracts has recently received more research attention [9,10]. Antioxidants incorporated into a film can, not only prevent oxidative damage in fatty foods, but also act as a functional additive by migrating from the packaging into the food product. However, the stability of polyphenols can deteriorate during food processing and storage because of their sensitivity to oxygen, light, and heat. Encapsulating phenolic extracts in various types of wall material can efficiently reduce this deterioration during the preparation of the films. Encapsulation can also help to regulate the kinetics of the active compounds as they are released into food products while maintaining the physical properties of the film.

Spray drying is the most widely used technique for encapsulating active and heat-labile compounds because of its short thermal contact time and suitability for industrial application. The type of wall material is also crucial when producing microencapsulates by spray drying [11]. Of the various types of wall material, conjugated mixtures of protein and polysaccharides, such as sodium caseinate and pectin, are of great interest because of their complementary effects on the stability of the core material [12,13]. Sodium caseinate is derived from casein, the principal protein in the milk of bovine and other ruminant animals. Its emulsifying properties combined with its heat stability promote its suitability as a wall material for encapsulation [14]. Pectin is a negatively charged plant polysaccharide which has a wide range of food applications including as a protective carrier because of its gel-forming and stabilization properties. In our recent study, polyphenols obtained from SBT were successfully encapsulated in a mixture of pectin and sodium caseinate to produce a functional food ingredient [8]. These encapsulated polyphenols can be used as stable active compounds in different food applications. However, few studies have reported on the application of these prepared functional microencapsulates to food products and active food packaging incorporating SBT extract microencapsulates has not yet been developed to the best of our knowledge. For producing food packaging, the use of biobased polymers rather than synthetic material is gaining popularity because they are biodegradable, cheap, abundant, and edible. Starch from a variety of plant sources is considered as one of the most promising biopolymers. In particular, cassava starch has been reported to be an excellent raw material for food packaging because of its characteristics of being odourless, tasteless, colourless, non-toxic, and with a high amylopectin content and high viscosity [15,16,17,18]. It has also been used as a carrier for antioxidants and antimicrobials in active food packaging and improved the physical properties of the film [19,20,21].

Hence, the present study aimed to develop a functional film incorporating SBT extract and to investigate the effect of incorporating free and encapsulated forms of the extract on the physico-chemical properties and migration behaviour of active compounds into food simulants. The performance of antioxidants in preventing lipid oxidation of soybean oil will also be studied.

## 2. Results and Discussion

### 2.1. Fourier Transform Infrared (FTIR) Analysis of Films and SBT Extract Powders

**Figure 1:**
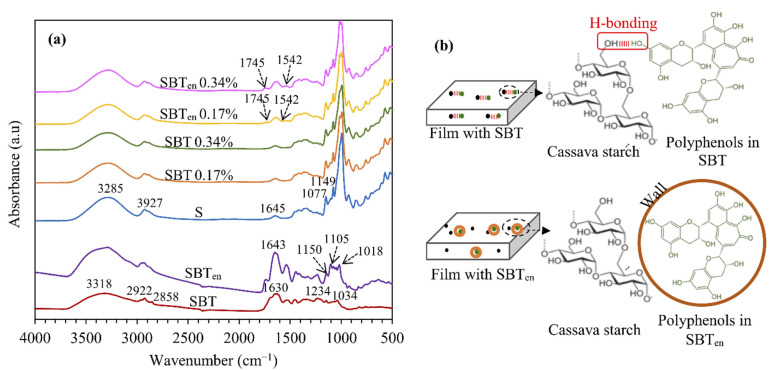
(**a**) FTIR spectra of spray-dried SBT and SBT_en_, starch-based films without (S), and with SBT or SBT_en_; (**b**) Schematic illustration of interaction between starch and SBT polyphenols in active films with SBT or SBT_en_.

FTIR spectroscopy is used for analysing the functional bonds and intermolecular interactions between compounds by identifying their molecular vibrations. The infrared spectra of free and encapsulated spent black tea extract powders (SBT, SBT_en_ respectively) are shown in Figure 1a. The FTIR spectrum of SBT extract reflects the main functional groups in polyphenols, amino acids, and alkaloids. The broad band at 3318 cm^−1^ is related to the O–H and N–H stretching modes in tea extract [22,23]. The peaks observed at 2922, 2858, and 1034 cm^−1^ have been attributed to C–H stretching, O–H stretching in alkanes and carboxylic acid, and C=C bond stretch in aromatic rings, respectively [24]. The 1234 cm^−1^ band probably arises from the C–O group in polyols such as hydroxyflavones and catechins [25]. The absorption bands at 1630 and 1530 cm^−1^ have been attributed to amide Ι (C=O stretching) and amide ΙΙ (N–H bending) in the amino acids present in black tea [26].

For SBT_en_ powder, a strong peak observed at 1643 cm^−1^ could be caused by the migration of the –COO stretching vibration at 1630 cm^−1^ from the spectrum of pectin and the C=O band at 1648 cm^−1^ from the spectrum of casein (Figure S1). These spectral changes have been documented as characteristics of the pectin–casein bonding which may be created due to the Maillard conjugation [27,28,29]. The results from X-ray diffraction and thermal stability in our previous study have also confirmed the formation of a pectin–caseinate complex [8]. Moreover, emerged new peaks at 1105 and 1018 cm^−1^ in SBT_en_ presented a band at 1150 cm^−1^ (C–O stretching) in both SBT and SBT_en_ and a mild shift in the peak of O–H stretching at 3290 cm^−1^, and indicated that conjugation had occurred between the polyphenols and sodium caseinate–pectin during the encapsulation process (Figure S1). This result was consistent with Jin et al.’s study [30], which revealed FTIR spectra of conjugation between tea polyphenols, pectin, and soy protein.

In the IR spectra of all films (Figure 1a), the wide band between 3000 and 3600 cm^−1^ associated with O–H stretching and the peak at 2927 cm^−1^ could be attributed to the C–H from alkyl groups [31]. The peaks at 1149 and 1077 cm^−1^ were attributed to C–O bond stretching of the C–O–H group. It has been reported that the vibrational band at around 1645 cm^−1^ is related to the O–H bending of the adsorbed water in the amorphous regions of cassava starch [32]. In films incorporating SBT_en_, this peak overlapped with C=O stretching (amide Ι; at 1635 cm^−1^) in the sodium caseinate wall materials. The films with SBT_en_ exhibited bands at 1542 cm^−1^ (amide ΙΙ) and 1745 cm^−1^, corresponding to typical peaks for SBT_en_ powder, thus confirming the successful incorporation of the encapsulated powder.

After adding intact SBT extract to the film, a flattening and a slight red shift of the O–H stretching band from 3285 cm^−1^ to 3291 cm^−1^ can be observed, indicating that there was no chemical interaction, but hydrogen bonds between active groups of the starch matrix and the phenolic hydroxy groups in the SBT. In contrast, the related peak in films with SBT_en_ showed less flattening than with SBT and shifted to the lower wavenumber. This behaviour indicated the higher availability of O–H in SBT_en_ films than in the other formulations owing to having unbound surface O–H groups in SBT_en_ film [33]. The ratio of the intensities of the peaks at 3300 and 1149 cm^−1^ (I_3300_/I_1149_), associates to the stretching vibration of ‘C–O’ in the ‘C–O–H’ group, was also calculated to compare the number of hydroxyl groups available in the different formulations. The films containing SBT_en_ exhibited higher ratios indicating a higher number of available hydroxyl groups and the film containing SBT showed a lower number than the other formulations [19]. It can thus be presumed that phenolic hydroxyl groups can interact with the cassava starch matrix by creating hydrogen bonds, but encapsulating SBT in wall materials can interrupt these bonds, as illustrated in Figure 1b.

### 2.2. Morphology of Control and Active Films

**Figure 2:**
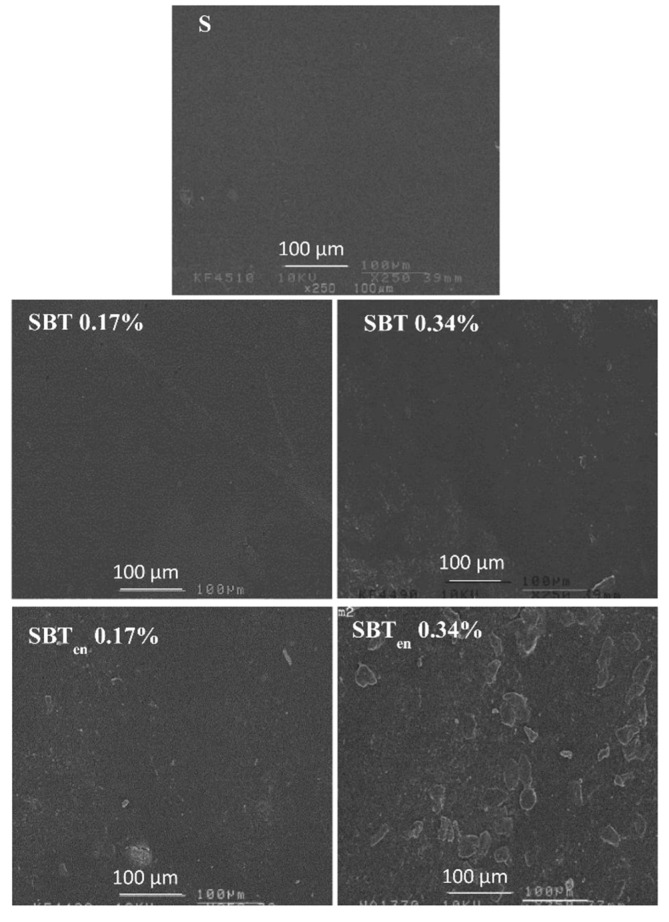
SEM micrographs of the surface of starch films incorporating free or encapsulated SBT extracts: control film (S) and active films incorporating SBT or SBT_en_ at 0.17% or 0.34%.

The effect of incorporating free and biopolymer-encapsulated SBT extract into starch films on their superficial morphology was analysed using scanning electron microscopy (SEM) images (Figure 2).

The cassava starch films exhibited a smooth, homogenous, and flat surface, but adding SBT generated the rough surface on films, which interfered with the regular starch matrix. This damaging of matrix could be ground for the poor mechanical properties of the active films. Films incorporating the free SBT extract showed no significant differences in their surfaces regardless of the polyphenol concentration, suggesting that the SBT extract had spread evenly throughout the starch matrix and interacted via hydrogen bonding during the processing of the film. Similar results have been observed during the development of active starch films incorporating tea polyphenols [34]. After adding SBT_en_, white particles were observed on the surface of the films, possibly related to the partial dissolving of SBT_en_ resulting in the entrapment of SBT extract within the core of the microencapsulates. The SBT_en_ 0.34% films exhibited a very rough surface with a less homogenous surface, suggesting that the polymer chains had been interrupted by the microcapsules. This phenomenon has also been reported where undissolved particles were observed on the surface of cassava starch films incorporating anthocyanin microencapsulates [35].

### 2.3. Tensile Properties, Thickness, Young’s Modulus, and Viscosity of Film-Forming Solutions

**Figure 3:**
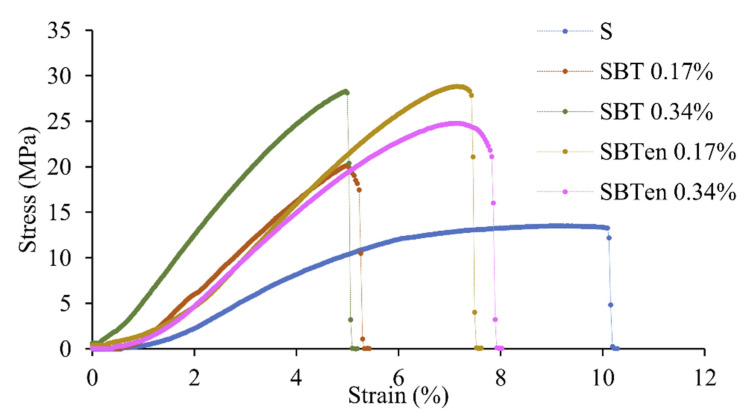
Stress–strain curves of starch films incorporating free or encapsulated SBT extracts: control film (S) and active films incorporating SBT or SBT_en_ at 0.17% or 0.34%.

The mechanical behaviour of films is related to their internal structure and can be described in terms of tensile strength (TS), elongation at break (EAB), Young’s modulus (YM), and film thickness. For the different film formulations, Table 1 shows the mean values of their mechanical parameters and Figure 3, their stress–strain curves.

Incorporating free and encapsulated SBT decreased the thickness of the active films associated with the decreasing starch mass. The control film with the highest mass of cassava starch exhibited the highest thickness influenced by the viscosity of the film-forming dispersion (FFD) (Table 1), an important parameter to be evaluated for the film casting process. This can affect the spread ability, thickness, uniformity of the casting layer, and the mechanical properties of the biopolymer film [36]. Table 1 shows that the tensile strength (TS) of the films incorporating SBT was significantly higher than that of the control film (*p* < 0.05) with the film incorporating 0.34% SBT exhibiting the highest value of 25.33 MPa. Simultaneously, the strain at break of the SBT films decreased significantly. This behaviour can be attributed to the generation of hydrogen bond interactions between the black tea extract and the cassava starch molecules. The spent black tea extract comprises polyphenols with hydroxyl groups, particularly thearubigins, theaflavin, and catechin, which influence their linking with the functional groups in cassava starch. Similar results have been found in starch films incorporating tea polyphenols and rosemary extracts [19,34]. The incorporation of encapsulated SBT into film also significantly increased both the value of TS and Young’s modulus (YM) (*p* < 0.05). This may have been caused by the microencapsulates acting as a reinforcement filler in the starch matrix, thus developing a less continuous microstructure. However, the values of TS and YM in films with SBT_en_ were lower than those with free SBT at 0.34%, possibly influenced by the sodium caseinate and pectin used as wall materials in the microencapsulates. High levels of rigidity and brittleness are seriously disadvantageous limitations in active biobased packaging films and thus affect their use in the food industry. Consequently, incorporating encapsulated SBT into the starch matrix can overcome these disadvantages by increasing the elasticity and positively affecting the mechanical behaviour of the film.

### 2.4. Water Vapour Transmission Rate

The water vapor transmission rate (WVTR) is a critical property of packaging films which affects the moisture level of the food products by preventing the loss or gaining moisture. The WVTR of the different film formulations with free and encapsulated SBT was assessed (Table 1). The results indicated that the significant effect on WVTR of adding SBT at 0.17% could be attributed to the formation of hydrogen bonds between the starch and polyphenols, as discussed previously. This can reduce the quantity of hydrophilic functional groups, thus lowering the WVTR value. However, this value increased again with the addition of SBT at a concentration of 0.34% owing to the hygroscopic nature of phenolic extracts. The WVTR of a film can be influenced by several factors such as the hydrophobic/hydrophilic nature of the materials used for the preparation of film, the presence of cracks or voids, and steric hindrance and tortuosity in the structure [37]. The WVTR values of active films with encapsulated SBT increased, but not above those of the control and the SBT 0.34% films, possibly because of the water-soluble nature of pectin in an aqueous environment [38] and the hydrophilic groups of sodium caseinate in the wall of the microencapsulates [39]. However, the decrease in the WVTR indicated the adequate cohesion of the polymeric chain formed, thus illustrating that strong intermolecular interactions had created barriers to the diffusion of water vapor through the matrix, whereas the presence of microcapsules as fillers can also create a tortuous path for water molecules to pass through.

### 2.5. Light Transmission and Transparency of Films

**Figure 4:**
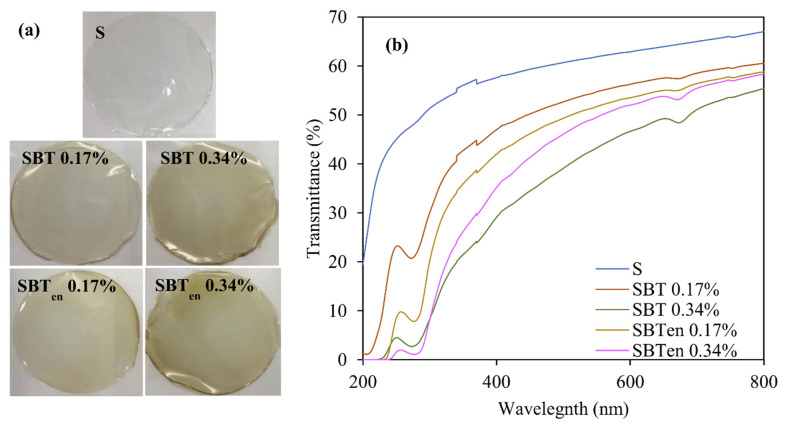
The visual appearance (**a**) and light transmittance (**b**) of the control film (S) and starch films incorporating free or encapsulated SBT extracts at 0.17% or 0.34%.

The light transparency of packaging materials is an important parameter, because it affects the quality of the protection to foods while also influencing its appearance and attractiveness. Figure 4a shows that the control film based only on cassava starch was clear and transparent, but adding SBT or SBT_en_ made the films slightly brown in colour. The control film (S) exhibited the highest transparency value (−1.783), but after adding the SBT and SBT_en_ powder, the transparency of the resulting active films (average: −2.98 and −3.15, respectively) decreased significantly (*p* < 0.05).

Light can catalyse many reactions causing food to deteriorate, influencing lipid oxidation, off-flavour development, the generation of undesired colours, and the degradation of nutritional compounds. UV light, with a higher energy than visible light, has a great potential for breaking chemical bonds. Therefore, blocking out UV light is a desirable property for a packaging film, particularly when used on foods with a high lipid content which are prone to photosensitized oxidation.

The wavelength of ultraviolet (UV) light ranges from 10 to 380 nm and visible light from 400 to 780 nm [40]; thus, the transmission of UV and visible light was determined at a wavelength between and 200 to 800 nm (Figure 4b). All the active films provided a lower transmission of UV light than the control film (S), possibly due to the fact that UV light was absorbed after incorporating the phenolic extracts into the starch matrix. It was shown to increase the obstruction to UV light with increasing SBT or SBT_en_ content. This behaviour agreed with previous studies on active cassava starch film incorporating Chinese berry anthocyanins [21], and cassava starch/chitosan active film with Pitanga leaf extract [41]. In the case of the SBT_en_ 0.34% film, the light transmission in the range of 400–800 nm was 35–58%, whereas for the SBT 0.34% film, it was 29–55%, possibly because the polymeric wall materials in SBT_en_ had covered the active compounds.

### 2.6. Antioxidant Content of Films and Their Migration into Food Simulants

Biopolymer films incorporating antioxidant compounds are manufactured for active food packaging applications. The incorporated antioxidant compounds can help reduce oxidative reactions in foods and thus significantly increase their shelf life. The DPPH radical scavenging activity assay is commonly used to evaluate the activity of antioxidant compounds in food by quantifying their ability to quench the DPPH radical. The dark purple colour of DPPH disappears when it is reduced to its non-radical form. The degree of fading of the free radical scavenger can be quantified by measuring the absorbance at 517 nm. The antiradical effects of all active film formulations measured by DPPH are shown in Table 2. The results showed that the concentration of polyphenols and encapsulation significantly affected the antiradical effect of the films (*p* < 0.05). The total antioxidant activity of the SBT_en_ 0.34% film was the highest at 630 µg GAE/g film, whereas that of the SBT 0.17% film was significantly lower than that of the other formulations at 173 µg GAE/g film. This can be attributed to the loss of antioxidant polyphenols during the preparation and drying of films incorporating free SBT. However, the microencapsulation of polyphenols could preserve the antioxidant activity of the films, which agreed with a report on the use of microencapsulation for preserving green tea polyphenols during different food applications [42]. Incorporating a higher level of polyphenols (0.34%), whether encapsulated or free, also significantly increased the antioxidant activity of the films.

**Figure 5:**
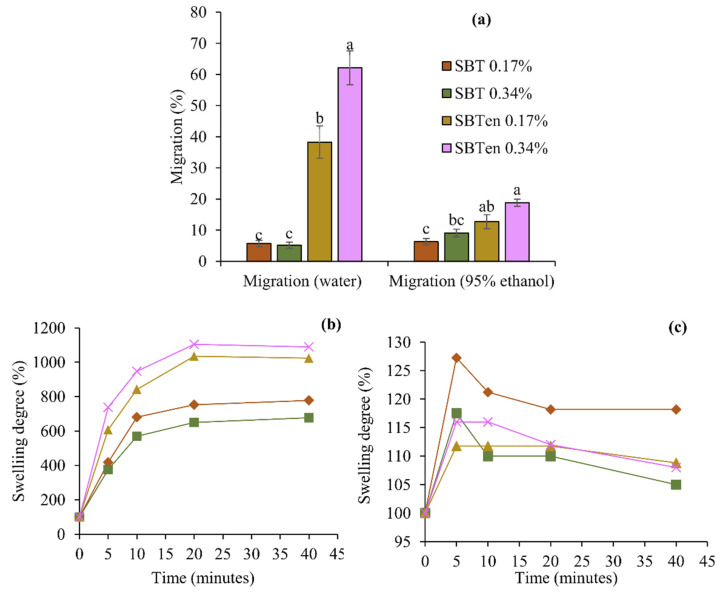
Migration percentage of antioxidant compounds into water and 95% ethanol, (**a**) Swelling degree in water (**b**) and Swelling degree in 95% ethanol (**c**) of starch films incorporating free or encapsulated SBT extracts at 0.17% or 0.34%. Different letters in each food simulant (a–c) indicated a statistically significant difference (*p* < 0.05).

The migration or release test is important for providing information on the affinity of food products for active materials, thus making it feasible to select the most suitable active material for each type of food. The release completely depends on the compatibility of the antioxidant compound with the food product or food simulant [43]. Various studies have quantified the compounds which are released into recommended food simulants such as water (representing aqueous foods) and 95% ethanol (representing fatty foods) [44,45]. The results of the present study showed a significantly higher release of antioxidant compounds from films incorporating SBT microcapsules (SBT_en_) into water than into 95% ethanol (Table 2, Figure 5a).

The three main factors affecting the migration of active compounds into food simulants are the liquid diffusion into the film network, the solubility of the film in the simulant, and the diffusion of the film into the simulant [44]. The liquid diffusion into the film matrix also depends on the polarity of the simulant, as indicated by the swelling degree of the film. Figure 5b,c shows that the swelling degree of all the active films in water was higher than those in ethanol and that the films incorporating encapsulated SBT exhibited a higher swelling degree in water than those incorporating free SBT. This could be the effect of the hydrophilic nature of the films, thus making them swell more in water than in ethanol. In particular, films incorporated the encapsulated SBT were more hydrophilic than the film incorporated free SBT, and thus led to the higher migration of antioxidant compounds into water. In contrast, films with free SBT released more active compounds into 95% ethanol than into water, possibly because of the greater affinity and swelling of SBT films in 95% ethanol (Figure 5c). This could also be attributed to the hydrophobic nature of polyphenols in their free form, but when covered with a polymer wall material, the resulting microcapsules exhibited a more hydrophilic nature. Even though the release from SBT films into 95% ethanol was higher than into water, it was always lower than that from the SBT_en_ films. This behaviour could also be attributed to the presence of hydrogen bonds between the polyphenols and starch molecules in the SBT film, but their absence in the SBT_en_ films promotes the release of microcapsules. Thus, active films incorporating encapsulated SBT released more into both types of food simulant, but after release, the hydrophilic nature of the microcapsules seemed to limit the complete release of active compounds more into ethanol than into water. A similar observation has been reported for the release of microencapsulated eugenol from thermoprocessed starch films [46].

### 2.7. Effect of Antioxidant Activity on Preventing Lipid Oxidation

**Figure 6:**
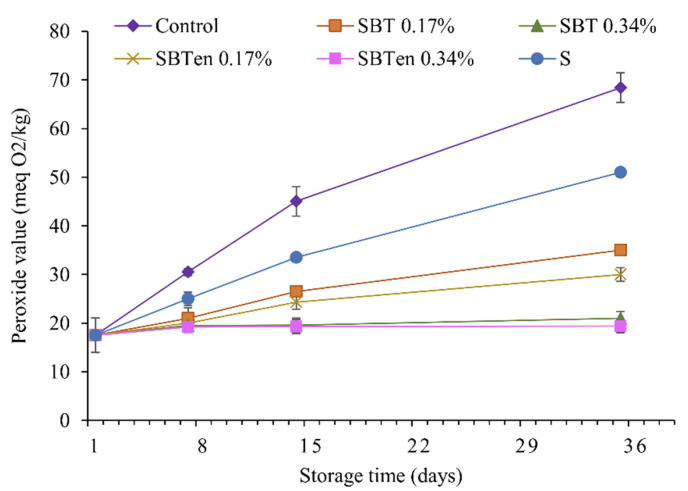
The changes of peroxide value of soybean oil during 35 days of storage at 27 °C, open glass vial (control) and starch films without and with incorporating free or encapsulated SBT extracts at 0.17% or 0.34%.

Lipid oxidation is a detrimental process in food systems, because it can reduce the nutritional value and sensory quality of foods and produces toxic compounds hazardous to human health. The degree of lipid oxidation is commonly measured by evaluating the peroxide value (PV) of foods. This is also an important test for measuring the effectiveness of active packaging films placed in direct contact with foods. The PV determines the concentration of hydroperoxide, the primary oxidation products of foods, with a high peroxide value indicating a higher level of lipid oxidation or food spoilage [47]. In the present study, the PV of soybean oil samples in contact with the different formulations of films was measured during storage for 35 d. The soybean oil in the open vial reached the highest PV of 68.4 ± 3.04 (meq O2/Kg) after 35 d of storage (Figure 6) because of its exposure to light and oxygen.

The all-active films exhibited a lower PV than the open control sample and the sample sealed with the starch film. As the concentration of polyphenols increased, the PV of the oil decreased. This suggested that adding SBT extract to the film in either the free or encapsulated form could retard the formation of peroxides due to lipid oxidation. Theoretically, lipid oxidation can be induced by oxygen in the presence of initiators such as heat, free radicals, light, and metal ions [48]. Thus, the polyphenols in the SBT extract could scavenge the free radicals in oil by donating hydrogen from the phenolic hydroxyl group, which helped to stop the chain of radical propagation.

The PV of oil sealed with SBT_en_ films was lower than that of oil sealed with SBT films, possibly because of its higher release of antioxidant compounds. However, the PV of oil samples sealed with either SBT or SBT_en_ films at 0.34% were similar, possibly because the lower transparency of the SBT films at 0.34% would remove the initiators needed for oil oxidation.

## 3. Materials and Methods

### 3.1. Materials

The cassava starch was obtained from a local supermarket in Sapporo (Hokkaido, Japan). Unblended black tea was supplied by New Vithanakande Tea Factory (Pvt) Ltd., (Ratnapura, Sri Lanka). Glycerol, gallic acid, 2,2-diphenyl-1-picrylhydrazyl (DPPH), Folin and Ciocalteu phenol reagent, and casein sodium salt from bovine milk were provided by Sigma-Aldrich (St. Louis, MO, USA), and pectin from citrus, sodium carbonate, magnesium nitrate, ethanol, and methanol were provided by Fujifilm Wako Pure Chemical Corp. (Osaka, Japan). Distilled water was used in all experiments. All other chemicals and solvents used were of analytical grade.

### 3.2. Preparation of SBT Powders

#### 3.2.1. Preparation of SBT

The SBT was produced as described previously [8]. Briefly, black tea leaves were brewed in boiling water for 6 min (2 g/100 mL), then filtered to obtain the residue. The SBT was prepared after air-drying the filtered residue in an oven at 45 °C.

#### 3.2.2. Extraction and Encapsulation of SBT Polyphenols

The antioxidant phenolic compounds from SBT were extracted by subcritical solvent extraction (SSE) using the conditions optimized in a previous study [8]. Briefly, a mixture of pure water and ethanol (71% concentration) and SBT (20 mL/g) were mixed in an 11-mL reactor with an agitator (Chemi-station PPV 3000, Tokyo Rikakikai Co. Ltd., Tokyo, Japan). The extraction reactor was purged with nitrogen gas and 2.0 MPa of initial pressure was applied. The sample in the reactor was then heated to 180 °C and maintained there for 10 min. During extraction, the agitation speed was kept at 1000 rpm to prevent any local overheating and to increase the mass transfer. After the extraction, the reactor was cooled in a cold-water bath; then, the sample was filtered through filter paper (6 μm) to recover the SBT extract. The recovered SBT extract was concentrated using a rotary evaporator (N-1210 and SB-1300 water bath, EYELA Tokyo Rikakikai Co., Ltd., Tokyo, Japan), then stored at 4 °C until encapsulation.

The SBT extract was microencapsulated as described previously [8]. A 50%:50% combination of pectin and sodium caseinate was used as the wall or coating material. First, the coating material was dissolved in distilled water at 90 °C (3 g/100 mL), then stirred until a clear dispersion was obtained. The polymer solution was then kept in a refrigerator overnight to allow complete hydration. On the next day, the concentrated SBT extract was added dropwise to the prepared biopolymer solution (1 g SBT extract: 20 g solution) after it had been heated to 40 °C; then, the mixed solution was stirred for 20 min. The prepared feed solutions were sonicated for 20 min, then homogenized for 30 min (HERACLES-16g, Koike Precision Instruments, Tokyo, Japan). The samples with a 3% solids concentration were then spray-dried with a laboratory-scale spray dryer OSK 55MO102 (Osaka Seimitsu Kikai Co. Ltd., Osaka, Japan) using a spray nozzle (diameter 0.5 mm), an inlet air temperature of 140 °C, an outlet air temperature of 85 °C, an atomization pressure of 0.4 MPa, and a feed flow rate of 5 mL/min. The non-encapsulated SBT extract was spray dried under similar conditions to the other samples with each experiment performed in duplicate. The resulting SBT extract powders with or without encapsulation were packed in zip-lock bags, covered with aluminium foil, then stored in a refrigerator until further use.

### 3.3. Preparation of the Films

The films were prepared by the solvent casting process, as described in previous studies [19,35,46]. Cassava starch control films were produced by blending 4.0 g of starch, 1.2 g of glycerol, and 84.0 g of distilled water. Active starch films were prepared by replacing the starch component in the formulations with the same amount of free or encapsulated SBT extract powder with predetermined phenolic content by Folin–Ciocalteu method [8] (total phenolics content of powders: 359.90 and 17.14 µg of gallic acid equivalent/mg, respectively), to obtain films with two different concentrations of total polyphenols (Table 3). The total solids concentration of all the control and active films were set at 4% (*m*/*m*). First, an aqueous starch dispersion was heated at 95 °C for 30 min under constant stirring until the starch was gelatinized. Glycerol was then added to the solution at a total solid: glycerol ratio of 10:3 (*m*/*m*) and agitated for 30 min. After cooling the solutions to 40 °C in a cold-water bath, the free and microencapsulates of SBT were added to the starch solution, then homogenized for 15 min. The film-forming dispersions (FFD) obtained were degassed by sonication then poured into polypropylene Petri dishes while maintaining the amount of FFD in the dish constant at 0.28 g/cm^2^. After drying at 35 °C for 24 h, the films were conditioned at 25 °C and 53% relative humidity over saturated Mg (NO_3_)_2_ before characterization.

### 3.4. Measurement of Viscosity of FFD

The viscosity of all the FFD was measured using a Sinewave Vibro Viscometer SV-10 (A&D Co. Ltd., Tokyo, Japan). All measurements were carried out at room temperature in triplicate, and the average value was taken as the final value.

### 3.5. Characterization of the Films

#### 3.5.1. Fourier Transform Infrared (FT-IR) Spectroscopy of Powders and Films

The preliminary structures of the SBT and SBT_en_ powders were characterized using an FTIR spectrophotometer (JASCO FTIR660plus, JASCO Corp., Tokyo, Japan). The samples were prepared by pressing a mixture of the powder sample and KBr into pellets. The functional groups of the films were identified by the FTIR spectrophotometer equipped with an attenuated reflection accessory, ATR (Spectrum 100, PerkinElmer Co., Ltd., Shelton, CT, USA). A spectral resolution of 4 cm^−1^ was used and 32 scans were acquired for each spectrum in the range of 650–4000 cm^−1^. This experiment was performed in a room at 23 °C and 50% relative humidity.

#### 3.5.2. Scanning Electron Microscopy (SEM)

The surface morphology of the films was observed using a field emission scanning electron microscope (SEM, JSM-6301F, JEOL Ltd., Tokyo, Japan). The samples were dried at 35 °C for 12 h, mounted on aluminium stubs using double-sided carbon tape, then sputter-coated with gold. The micrographs were captured at an accelerating voltage of 10 kV.

#### 3.5.3. Film Thickness and Tensile Properties

The thickness of the films was measured by a digital micrometer (ABSOLUTE Digimatic Micrometer, Mitutoyo Corp., Kawasaki, Japan) to the nearest 0.001 mm. The average thickness was based on measurements at three random positions on the film.

The tensile properties, tensile strength (TS), elongation at break (%EAB), and Young’s modulus (YM) of the films were analysed using a universal testing machine system (AG-100kNXplus, Shimadzu Corp., Kyoto, Japan) according to the ISO 527-3 (2018) test method [49]. All the films were initially cut into a dumbbell shape using a cutter (JIS K7113 No.1½, Shimadzu Corp.) and preconditioned for 5 d at 23 °C and 50% relative humidity before the mechanical testing. The initial grip separation and the testing speed were set at 50 mm and 50 mm/min, respectively. The results are based on three measurements for each formulation.

#### 3.5.4. Water Vapour Transmission Rate (WVTR)

The water vapour transmission rate of the samples was analysed as described previously [50], with minor modifications. First, the film samples were equilibrated in a desiccator containing a saturated solution of NaCl (75% relative humidity). The films were then fixed using elastic bands over the top of a weighing bottle containing 5.0 g of anhydrous CaCl_2_, then weighed immediately (W_1_). All the bottles were then placed in a chamber for 24 h (75% relative humidity at 25 °C) then weighed again (W_2_). The WVTR was calculated as follows:

(1) $$\mathrm{WVTR}\;(g\;\mathrm{mm}/m^{2}\;24\;h)=\frac{\left(W2-W1\right)\times \;\mathrm{Film}\;\mathrm{thickness}}{\mathrm{Area}\;\times \mathrm{Holding}\;\mathrm{time}}$$

#### 3.5.5. Light Transmission

The light transmission of the films was measured using a UV-Vis spectrophotometer (JASCO V-560, JASCO Corp., Tokyo, Japan) in the wavelength range of 200–800 nm. First, the films were cut into rectangular strips (3.5 cm × 0.5 cm), then placed directly into a cuvette to allow the light beam to pass through the films. The analyses were performed in triplicate and an empty cell was used as the reference. The film transparency for visible light was determined at 600 nm [51]. The percentage transparency was calculated as follows:

Transparency value = (log T_600_)/x (2)

where T is the fractional transmission at 600 nm and x is the thickness of the film sample (mm).

#### 3.5.6. DPPH Radical Scavenging Assay

The antiradical effect of the films was determined by the 2,2-diphenyl-1-picrylhydrazyl (DPPH) scavenging activity method [52] with slight modifications. First, a piece of film (30 mg) was completely dissolved in distilled water, then 3 mL of ethanol was added. The resulting solution was centrifuged at 6000× *g* for 10 min. Two millilitres of the supernatant and 5 mL of 0.1 mM DPPH solution were mixed, kept in the dark for 30 min, then the absorbance was measured at 517 nm using a UV–vis spectrophotometer (JASCO V-560, JASCO Corp., Tokyo, Japan). Gallic acid was used as the standard for preparation of the standard curve (0.5–6.0 μg/mL, R^2^ = 0.99). The DPPH scavenging capacity was expressed as micrograms of gallic acid equivalent/g (dry weight) film (µg GAE)/g film).

#### 3.5.7. Migration Test

Pieces of film sample (2 cm × 2 cm) were placed in 8 mL of water (representing an aqueous food) and 95% ethanol (representing a fatty food) in separate glass vials. The vials were then purged with nitrogen gas before closing and kept at 25 °C for 7 d. The migration of the antioxidant compounds into each food simulant was then tested using the DPPH method, as described earlier (Section 3.5.6). The results were expressed as micrograms of gallic acid equivalent/g (dry weight) film (µg GAE)/g film).

#### 3.5.8. Peroxide Value (PV) of Soybean Oil

Soybean oil (5 mL) was poured into dark glass vials, then sealed with films of each formulation (S, SBT 0.17%, SBT 0.34%, SBT_en_ 0.17% and SBT_en_ 0.34%). The sealed vials containing the oil were then placed upside-down to ensure the oil was in contact with the films. An open dark glass vial containing 5 mL of soybean oil was used as a control. All vials were stored at 27 ± 2 °C and 50 ± 5% relative humidity under fluorescent light, then samples were taken after 0, 7, 14, and 35 d of storage to determine the PV of the oil. The peroxide value of the soybean oil was determined by iodometric titration. The oil sample (1 g) was mixed with 10 mL of glacial acetic acid and chloroform (3:2, *v*/*v*) in an Erlenmeyer flask. The mixture was shaken vigorously to dissolve the oil completely, then mixed with 0.5 mL of saturated potassium iodide solution. The mixture was then kept in the dark for 1 min before dilution with 30 mL of distilled water and titrated against 0.01 M sodium thiosulphate in the presence of a starch indicator (5 mL). The volume of sodium thiosulphate consumed was recorded and the PV expressed as millimolar equivalents of free iodine per kilogram of oil (meq of oxygen/kg). All the analyses were performed in triplicate.

### 3.6. Statistical Analysis

Statistical analyses were carried out using Minitab 19.1.1. (Minitab Inc., State College, PA, USA) to determine the significance of differences of factors and levels. One-way analysis of variance (ANOVA) and Tukey′s multiple comparison test were used to compare and identify significant differences (*p* < 0.05) between group means. The results were reported as the mean value of three repeated experimental data.

## 4. Conclusions

In the present study, a functional film has been developed using free and microencapsulated SBT extract as the active ingredient. The results showed that adding SBT either in the free or encapsulated form significantly affected the mechanical, barrier (water and light), and antioxidant properties of the films. Non-encapsulated SBT extract could modify the starch matrix by forming hydrogen bonds with cassava starch, thus inducing a reduction in the mechanical properties of the film and in antioxidant migration from the film. The film incorporating microencapsulated SBT extract exhibited a heterogenous film surface and enhanced physical properties compared with the SBT film. The microencapsulation of SBT protected the antioxidant compounds during the film processing and caused a significant increase in the migration of the active compounds into both the aqueous and fatty food simulants. The SBT_en_ 0.34% film exhibited the highest free radical scavenging activity and prevented lipid oxidation of soybean oil samples for more than 35 d.

## Figures and Tables

**Table 1:** Viscosity of FFD, thickness, mechanical properties, and water vapor transmission rates (WVTR) of starch films incorporating free or encapsulated SBT extracts.

Film	Viscosity of FFD (mPa.s)	Thickness (mm)	Tensile Strength (MPa)	Young’s Modulus (MPa)	WVTR (g mm^2^/m^2^ 24 h)
S	195.5 ± 2.5 ^a^	0.1133 ± 0.0036 ^a^	13.43 ± 0.141 ^b^	224.45 ± 86.3 ^c^	0.61 ± 0.19 ^a^
SBT 0.17%	119.0 ± 5.0 ^b^	0.1002 ± 0.0120 ^ab^	17.54 ± 4.535 ^ab^	231.94 ± 63.7 ^c^	0.29 ± 0.04 ^b^
SBT 0.34%	104.3 ± 5.1 ^bc^	0.0957 ± 0.0028 ^ab^	25.33 ± 3.706 ^a^	1282.39 ± 84.4 ^a^	0.54 ± 0.12 ^a^
SBT_en_ 0.17%	90.5 ± 1.5 ^bc^	0.0906 ± 0.0043 ^b^	25.17 ± 4.578 ^a^	955.05 ± 74.0 ^b^	0.38 ± 0.05 ^ab^
SBT_en_ 0.34%	82.5 ± 3.5 ^c^	0.0862 ± 0.0032 ^b^	23.47 ± 5.301 ^ab^	1088.19 ± 72.34 ^ab^	0.52 ± 0.13 ^a^

Starch films incorporating free or encapsulated SBT extracts: control film (S) and active films incorporating SBT or SBT_en_ at 0.17% or 0.34%. Different letters in the same column (a–c) indicate a statistically significant difference (*p* < 0.05) between mean values. Values represent the mean ± standard deviation of three individual runs.

**Table 2:** Antioxidant content and their migration into food simulants (water and 95% ethanol), expressed in terms of μg (gallic acid equivalent; GAE)/g film.

Film	Total Antioxidantμg (GAE)/g Film	Migration (Water) μg (GAE)/g Film	Migration (95% Ethanol) Μg (GAE)/gFilm
SBT 0.17%	173.14 ± 6.88 ^d^	9.84 ± 3.00 ^c^	10.85 ± 7.22 ^c^
SBT 0.34%	587.06 ± 6.98 ^b^	30.03 ± 4.00 ^c^	53.31 ± 5.11 ^b^
SBT_en_ 0.17%	276.13 ± 6.88 ^c^	105.63 ± 10.11 ^b^	35.08 ± 4.44 ^bc^
SBT_en_ 0.34%	629.70 ± 20.80 ^a^	391.22 ± 24.40 ^a^	118.53 ± 5.12 ^a^

Starch films incorporating free or encapsulated SBT extracts: control film (S) and active films incorporating SBT or SBT_en_ at 0.17% or 0.34%. Different letters in the same column (a–d) indicate a statistically significant difference (*p* < 0.05) between mean values. Values represent the mean ± standard deviation of three individual runs.

**Table 3:** Formulations of starch films incorporating free or encapsulated SBT extracts.

	Types	Polyphenols %	Starch (g)	SBT/SBT_en_ (g)	Glycerol (g)	Water (g)
Control films	S		4.000	-	1.2	84.0
Films with SBT extract	SBT 0.17%	0.17%	3.981	0.0190	1.2	84.0
	SBT 0.34%	0.34%	3.962	0.0380	1.2	84.0
Films with encapsulatedSBT extract	SBT_en_ 0.17%	0.17%	3.600	0.4000	1.2	84.0
	SBT_en_ 0.34%	0.34%	3.200	0.8000	1.2	84.0

## Data Availability

Data of the compounds are available from the authors.

## Supplementary Materials

The following are available online, Figure S1: FTIR spectra of spray dried SBT, SBT_en_, sodium caseinate, and pectin.

## Sample Availability

Samples of the compounds are not available from the authors.
