# Supplementary material for: Development and Characterization of Functional Starch-Based Films Incorporating Free or Microencapsulated Spent Black Tea Extract
Source: Molecules. 2021 Jun 25;26(13):3898. doi: 10.3390/molecules26133898 (PMC8271635; doi:10.3390/molecules26133898)
Supplement: Supplementary file 1 [file molecules-26-03898-s001.zip › molecules-1267985-supplementary.pdf]

**Supplementary material**

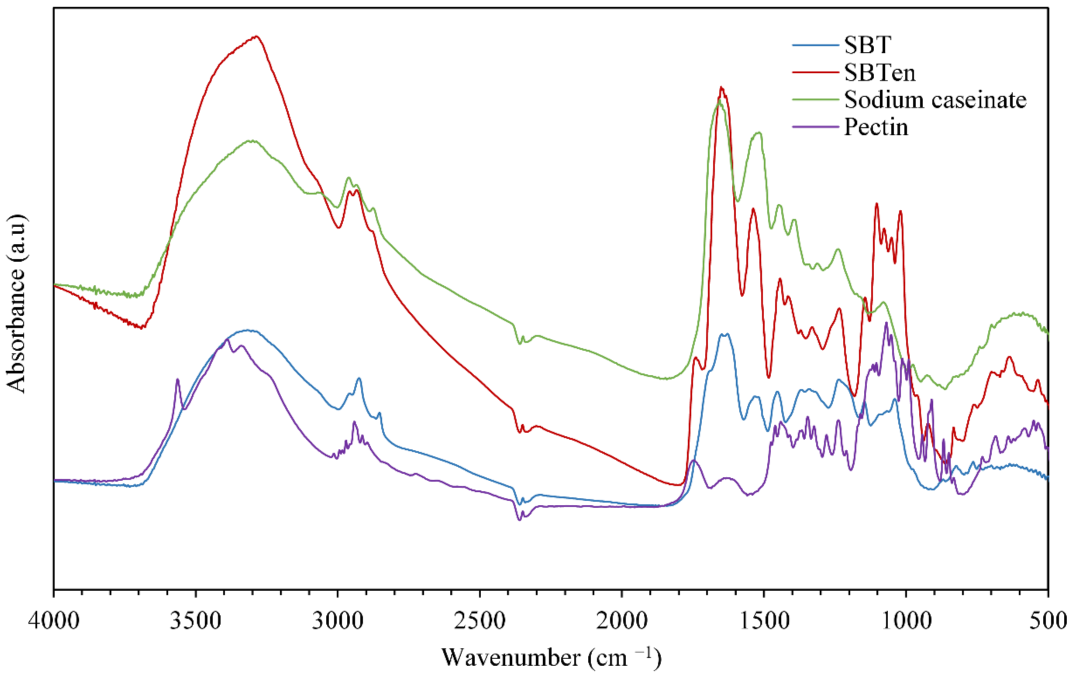


**Figure S1.** FTIR spectra of SBT, $SBT_{en}$, sodium caseinate and pectin powers.
